# Validity and Reliability of the Stress and Anxiety to Viral Epidemics-6 (SAVE-6) Scale to Measure Viral Anxiety of Healthcare Workers in Spain During the COVID-19 Pandemic

**DOI:** 10.3389/fpsyt.2021.796225

**Published:** 2022-02-01

**Authors:** Marta Moraleda-Cibrián, Oli Ahmed, Javier Albares-Tendero, Seockhoon Chung

**Affiliations:** ^1^Sleep Disorders Center, Centro Médico Teknon, Barcelona, Spain; ^2^Department of Psychology, University of Chittagong, Chattogram, Bangladesh; ^3^Research School of Population Health, Australian National University, Canberra, ACT, Australia; ^4^Department of Psychiatry, Asan Medical Center, University of Ulsan College of Medicine, Seoul, South Korea

**Keywords:** health personnel, SAVE-6, COVID-19, anxiety, stress

## Abstract

This study examined the validity and applicability of the Spanish version of the Stress and Anxiety to Viral Epidemics-6 items (SAVE-6) scale, which can be usually applied to the general population, to healthcare workers to briefly measure their anxiety responses to the viral epidemic. A total of 135 healthcare workers participated in this online survey from January to July 2021. Participants' sociodemographic characteristics were gathered, and their psychiatric symptoms were rated using SAVE-6, Goldberg Anxiety and Depression Scale (GDAS), and the Pittsburgh Sleep Quality Index (PSQI). The confirmatory factor analysis was conducted to examine the validity of the scales. The single-structure model of the SAVE-6 scale was adopted based on the results of the parallel analysis. We decided on the SAVE-6 scale, as it proved to be a good fit to measure healthcare workers' anxiety response to the viral epidemic. SAVE-6 showed good internal consistency (Cronbach's alpha = 0.827 and McDonald's omega = 0.834) and good convergent validity with Goldberg anxiety (*r* = 0.434, *p* < 0.001) and depression (*r* = 0.193, *p* = 0.043) scores, and PSQI score (*r* = 0.262, *p* = 0.002). The Spanish version of SAVE-6 is a reliable and valid rating scale to assess the anxiety response of healthcare workers specifically to the viral epidemic as a brief measure during the COVID-19 pandemic.

## Introduction

The COVID-19 pandemic has caused a challenging situation worldwide with a major health impact on vulnerable populations and populations with high risk for COVID-19 infection, such as healthcare workers. In Spain, the first confirmed case was reported in the Canary Islands on 31 January 2020, about two months after the first case was reported in China (1). The incidence and mortality of COVID-19 have been high in Spain despite a recognized, well-established health system (2). Socio-economic factors such as an aging population, reduced healthcare investment, fewer beds in intensive care compared to other countries of Europe, and even cultural factors may have played a role in the development of the COVID-19 pandemic in Spain (3).

Healthcare workers in this pandemic suffer from psychological problems such as severe work-related stress, burnout, depression, anxiety, or post-traumatic stress (4–6). The viral epidemic disaster is different from other disasters in that the victim of a viral disaster directly transfers the negative impact of the disaster to the front-line healthcare workers in the hospital. Healthcare workers are concerned about being infected while taking care of infected or high risk patients and about spreading the viral infection to their friends or family members (7, 8). The high percentage of COVID-19 infections among Spanish health professionals is associated with increased mental health symptoms investigated during the first wave of the COVID-19 pandemic. Healthcare staff has a 12 times higher risk of COVID-19 infection than the general population (9). Spanish healthcare workers reported that the frequency of anxiety, depression, acute stress, posttraumatic stress disorder, and insomnia varies between 30 and almost 60% (9, 10). The rate of acute mental health symptoms is a cause of concern due to the potential short and long-term work, personal, and social impact.

Therefore, specifically assessing healthcare workers' anxiety responses to the viral epidemic to manage their mental health should be considered important. For that, Chung et al. (11) developed the Stress and Anxiety to Viral Epidemics-9 items (SAVE-9) scale to measure healthcare workers' work-related stress and anxiety responses during the pandemic. It is a 9-item self-rating scale and validated in Korean (11), Russian (12), Italian (13), Japanese (14), Turkish (15), and German (16). The SAVE-6 scale, derived from the SAVE-9 scale, used to assess the anxiety response of the general population, was validated in Korean (17), Lebanese (18), and American samples (19). It was also examined for applicability to a particular population such as medical students (20), public workers (21), or cancer patients (22).

Originally, the SAVE-9 scale was reported to be divided into two factors: factor I (viral anxiety, item 1, 2, 3, 4, 5, and 8) and factor II (work-related stress, item 6, 7, and 9). The factor structure can be different cross-culturally (23). There might be differences in the pandemic situation, language, or healthcare system environment among various countries. The factor structure of the Japanese, Italian, and Turkish versions of the SAVE-9 scale was similar to that of the original version; however, the Russian and German versions were different (12, 16). Originally we developed the SAVE-9 scale to measure work-related stress and viral anxiety *specifically* to the viral epidemic and *specifically* to healthcare workers. To compare the differences in the level of viral anxiety of healthcare workers with that of the general population across culture, groups, or workplace, we need to validate the SAVE-6 scale among the various population including even healthcare workers. In addition, the SAVE-6 can also be applied to healthcare workers as a brief measure for anxiety response in the current pandemic situation; however, there is no previous study on its applicability to healthcare workers.

In this study, we aimed to validate the Spanish version of the SAVE-6 scale to confirm its construct validity to measure the anxiety response of healthcare workers in Spain during the COVID-19 pandemic. The validity of the SAVE-6 scale was also examined to explore the applicability to healthcare workers as a sole brief measure of their anxiety response.

## Materials and Methods

### Participants and Procedure

This study was conducted via an online survey from January to July 2021, and it is part of an ongoing study conducted to assess the stress and sleep problems of healthcare workers in Spain. Data were collected by an online questionnaire throughout social networks, healthcare groups (Quirónsalud group, Catalan Institute of Oncology) and scientific societies (Catalan and Spanish Sleep Society). Inclusion criteria were healthcare professionals, aged 25–69 years, of both genders, physicians and nurses in charge of patients with COVID-19 infection, working in hospitals, health care centers for COVID-19 patients, primary health care centers, geriatrics, and convalescent centers. Exclusion criteria was age <25 and >69 years, not in charge of patients with COVID-19, and unable to complete the survey. An e-agreement consent was obtained from participants in all cases. The study protocol was approved by the Institutional Review Board of Grupo Hospitalario QuirónSalud-Cataluña (2020/52-MSU-TEK). In the first part of the study, it was estimated that the sample size necessary to demonstrate a statistically significant difference of 25% on stress and sleep quality in the group of healthcare staff in charge of the COVID-19 patients vs. 10% in the group of healthcare staff in charge of patients with other pathologies with an alpha value of 0.05 and a power of 0.90 resulted in *N* = 216 divided into two groups (108.21 healthcare staff for COVID-19 patient and 108.21 for non-COVID-19 patients). In this manuscript, only results regarding healthcare staff on charge of COVID-19 patients were included. Sample size estimation was done by the rule of 10:1 (24), the ideal ratio of respondents to items. The SAVE-9 scale has nine items, so we needed at least 90 samples. In this study, we aimed to gather 150 samples, and we finally collected 135 samples.

### Survey

#### Sociodemographic Data and COVID-19 Related Questions

Participants' sociodemographic characteristics such as age, gender, weight, height, living with a partner (married or cohabiting), profession (physician or nurse), and place of work during the pandemic were collected. Concerning COVID-19, we asked questions such as, “Do you consider health protection measures against COVID-19 infection adequate?” “Do you have symptoms of COVID infection?” or “Did any of your family members contract COVID-19 infection?”

### Symptom Assessment

#### Stress and Anxiety to Viral Epidemics-6 Items (SAVE-6)

The SAVE-6 scale was specifically developed to investigate anxiety to the viral pandemic (17). This scale was derived from factor I of the SAVE-9 scale (11) and developed to assess anxiety responses of the general population. The items are rated on a 5-point Likert scale: 0 (never), 1 (rarely), 2 (sometimes), 3 (often), and 4 (always). Among the Korean population (17), the SAVE-6 scale was shown to be reliable (Cronbach's alpha = 0.815) and valid in line with other existing rating scales. The appropriate cut-off point was explored to be ≥ 15 (sensitivity = 0.70, specificity = 0.60) in accordance with a mild degree of generalized anxiety (GAD-7 score of 5). We translated the English version of the SAVE-6 into Spanish and back-translated it into English. In this study, we applied the Spanish version of the SAVE-6 Scale on healthcare workers in Spain.

#### Goldberg Anxiety and Depression Scale (GDAS)

GADS is a rating scale widely used in primary care to screen depression and anxiety (25). It consists of nine items for depression and 9 items for anxiety. Assessment of anxiety was done as follows: if at least three questions among the first four questions were answered “yes,” responses of the other five questions were continuously measured. Positive screening for anxiety was considered if four or more answers were positive. An assessment of depression was as follows: if at least one question among the first four questions were answered “yes,” responses of the other five questions were continuously measured. Positive screening for depression was considered in those cases with at least two positive answers. It was reported to be a rating scale with a sensitivity of 83.1% and a specificity of 81.8%. For the current study, the Spanish version of GADS was used (26).

#### The Pittsburgh Sleep Quality Index (PSQI)

The PSQI is a rating scale design to investigate sleep quality (27). It consists of 19 items grouped into seven components, weighted equally from 0 to 3. The total score of PSQI is summed from the scores of seven components, and it ranges from 0 to 21. A higher score reflects poor sleep quality, and a global PSQI score > 5 is the cut-off point to discriminate between good sleepers and poor sleepers with 89.6% sensitivity and 86.5% specificity. In this study, we applied the Spanish version of the PSQI scale (28).

### Statistical Analysis

We conducted the principal component analysis (PCA) using principal axis factoring, to explore the factor structure of the Spanish version of the SAVE-6 scale. The skewness and kurtosis were checked to determine whether each item was distributed within normal limits based on the values within the range ± 2 (29). The Kaiser-Meyer-Olkin (KMO) value and Bartlett's sphericity test were assessed to check data suitability and sampling adequacy. A screening test and parallel analysis (30–32), based on the Minimum Rank Factor Analysis (MRFA) (33), with a 95 percentile threshold based on the polychoric correlations matrix, was conducted using the FACTOR 10.10.03. program (33) to determine the number of factors to retain for the Spanish version of the SAVE-6 scale. We compared the explained real-data eigenvalues to the 95th percentile of random eigenvalues, and we decided where the real-data eigenvalues exceeded the 95th percentile of the random eigenvalues. We conducted a bootstrap (2,000 samples) maximum-likelihood confirmatory factor analysis (CFA) for the Spanish version of the SAVE-6 scale to examine the construct validity and applicability. Satisfactory model fit was defined by a standardized root-mean-square residual (SRMR) value ≤ 0.05, root-mean-square-error of approximation (RMSEA) value ≤ 0.10, and comparative fit index (CFI) and Tucker Lewis index (TLI) values ≥ 0.90 (34, 35). Psychometric properties of the scale were also assessed through the Item Response Theory (IRT) approach (graded response model [GRM]). Before running the GRM, IRT assumptions—unidimensionality, local dependence, and monotonicity were assessed through the R package *mokken* version 3.0.6 and *mirt* version 1.34. GRM was also run through the R package *mirt* version 1.34. In GRM, item misfit information was checked through the S-χ^2^ value. GRM provides slope/ discriminating parameters and threshold/ difficulty parameters. The reliability and internal consistency of the scales were assessed using Cronbach's alpha and McDonald's omega. To explore the convergent validity, Pearson correlation analysis between SAVE scales and depression or anxiety subscale of Goldberg scale and PSQI scale. The SPSS version 21.0 (SPSS, Inc., Chicago, Illinois), JASP version 0.14.1.0 software (JASP Team, Amsterdam, Netherlands), and RStudio were also used for statistical analysis.

## Results

All 135 healthcare workers participated in this study (Table 1). Among participants, 83.0% were female, 67.4% were physicians, and their mean age was 42.2 ± 9.1 years old. To the questions related to COVID-19, 21.5% answered that they had experienced symptoms of COVID-19 infection, and 34.1% answered that their family members got infected with COVID-19. The proportion of participants who were rated as having anxiety (Goldberg anxiety score ≥ 4) or depression (Goldberg depression score ≥ 2) were 59.3% (*N* = 80) or 82.2% (*N* = 111). Participants reported medical conditions such as asthma (nine subjects) or hypothyroidism (nine subjects).

**Table 1:** Demographic characteristics of participants (*N* = 135).

**Variables**	**Mean ±SD, N (%)**
**Sex (female)**	112 (83.0%)
**Age (years old)**	42.2 ± 9.1
20–29	16 (11.9%)
30–39	32 (23.7%)
40–49	54 (40.0%)
50–62	33 (24.4%)
**Living with partner (Yes)**	104 (77.0%)
No children	55 (40.7%)
With children	93 (68.9%)
Dependent person	7 (5.2%)
**Career**	
Physician	91 (67.4%)
Nurse	44 (32.6%)
**Place of work**	
Hospital	120 (88.9%)
Primary care centers	12 (8.9%)
Other	3 (2.2%)
**COVID-19 questions**	
Do you have symptoms of COVID-19? (Yes)	29 (21.5%)
Do your family members have symptoms of COVID-19? (Yes)	46 (34.1%)
**Psychiatric symptoms**	
Depression (Goldberg depression score ≥ 2)	111 (82.2%)
Anxiety (Goldberg anxiety score ≥ 4)	80 (59.3%)

### Factor Structure and Psychometric Properties of the SAVE-6 Scale

**Figure 1:**
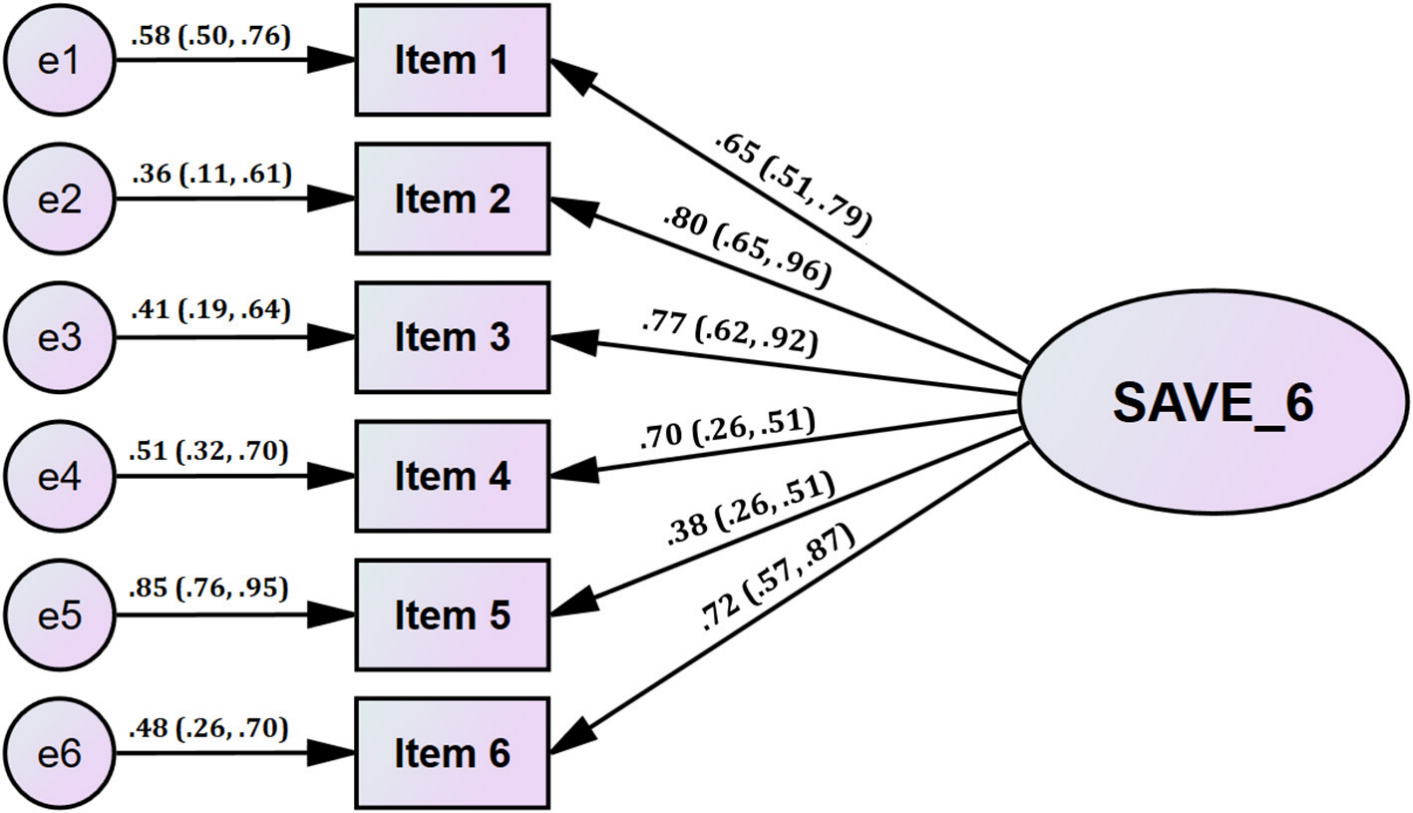
Factor structure of the SAVE-6 scale among healthcare workers in Spain.

The distribution of all six items of SAVE-6 was within the normal limit based on the skewness and kurtosis within the range of ± 2 (Table 2). The KMO measure of the SAVE-6 was 0.76, and Bartlett's test of sphericity showed a *p*-value of < 0.001, which means that this data was suitable for factor analyses. Parallel analysis using MRFA extraction advised the single-structure model of the SAVE-6 (real-data eigenvalue = 64.32, 95 percentiles of random eigenvalue = 34.33, Figure 1). In addition, the CFA of the SAVE-6 showed a good model fit (CFI = 1.00, TLI = 1.00, RMSEA = 0.00, SRMR = 0.057). Therefore, we can adopt the Spanish version of the SAVE-6 scale to assess the anxiety response of healthcare workers to the viral epidemic.

**Table 2:** Factor structure of the SAVE-6 scale and factor loadings.

**Items**	**Responses**					**Mean ±SD**	**Skewness**	**Kurtosis**	**CITC**	**CID**	**SAVE-6**
	**0**	**1**	**2**	**3**	**4**						
1. Are you concerned that the virus outbreak will continue indefinitely?	1.5%	13.3%	36.3%	34.1%	14.8%	2.47 ± 0.95	−0.134	−0.479	0.751	0.803	0.65
2. Are you concerned that your health will worsen because of the virus?	14.1%	24.4%	32.6%	19.3%	9.67%	1.86 ± 1.17	0.109	−0.750	0.738	0.777	0.80
3. Are you worried that you might get infected?	10.4%	24.4%	28.1%	23.7%	13.3%	2.05 ± 1.20	0.004	−0.897	0.739	0.785	0.77
4. Are you more sensitive to minor physical symptoms than usual?	15.6%	33.3%	20.0%	25.2%	5.9%	1.73 ± 1.17	0.185	−1.000	0.733	0.788	0.70
5. Are you worried that others might avoid you even after the infection risk has been minimized?	45.9%	26.7%	16.3%	6.7%	4.4%	0.97 ± 1.14	1.069	0.319	0.770	0.848	0.38
6. Do you think your colleagues would have more work if you were absent from a possible quarantine and might blame you?	31.9%	19.3%	17.0%	16.3%	15.6%	1.64 ± 1.46	0.318	−1.282	0.731	0.786	0.72

*SAVE-6, Stress and Anxiety to Viral Epidemic-6 items*.

*0 = never; 1 = rarely; 2 = sometimes; 3 = often; 4 = always*.

Supplementary Table 2 shows the psychometric properties of the SAVE-6 through the GRM (an IRT model for polytomous items). Before running the GRM, IRT assumptions (unidimensionality, local dependence, and monotonicity) were assessed (Supplementary Table 1). Loevinger's *H* coefficient (0.50; Table 3) suggest the unidimensionality of the scale. Non-significant *p*-values (adjusted for false discovery rate [FDR]) suggested the absence of local dependence. Supplementary Table 1 suggests there was no violation of monotonicity. These statistics about assumptions suggest that IRT models are applicable.

**Table 3:** Scale-level psychometric properties of the SAVE-6.

**Psychometric properties**	**SAVE-6**	**Suggested cut-off**
Floor effect	0%	15%
Ceiling effect	3%	15%
Mean inter-item correlation	0.444	Between 0.15 and 0.50
Cronbach's alpha	0.827	≥ 0.7
McDonald's omega	0.834	≥ 0.7
Split-half reliability (odd-even)	0.895	≥ 0.7
Standard error of measurement	2.08	Smaller than SD (6.42, 5.01)/2
Ferguson delta	0.99	≥ 0.9
Loevinger's *H-*coefficients	0.50	-
*Rho* coefficient	0.836	≥ 0.7
IRT reliability	0.880	≥ 0.7
**Model fits of confirmatory factor analysis**		
*χ^2^*(df, *p*-value), *χ^2^*/df	7.581 (9, 0.577),	Non-significant, <5
CFI	1.00	>0.95
TLI	1.00	>0.95
RMSEA [90% CI value] (*p*-value)	0.00 [0.00, 0.09], 0.781	<0.08
SRMR	0.057	<0.08

Supplementary Table 2 shows items' fit statistics (S-χ^2^ and *p*-values [adjusted for false discovery rate]) of items. These non-significant *p*-values suggested that these items belong to the full scale (SAVE-6). It demonstrates the slope/discrimination parameters (α) and threshold/difficulty parameters (b). Slope parameters ranged between 0.752 (item 5) and 3.254 (item 2) (mean = 2.127). The slope of item 5 is moderate, item 1 is high, and the rest are very high. These values suggested that all items provide reasonable discriminating information about stress and anxiety that the SAVE-6 assess. Results regarding threshold/ difficulty coefficients (*b*) suggest that a higher latent trait or theta is required to endorse items 4 and 5 compared to other items. On the other hand, a lower latent trait or theta is required to endorse item 6. In items 4 and 5, only b_1_ coefficients were negative, and the rest were positive. This suggests that the above- average level of the latent trait or theta is required to endorse Likert-type response options—from “sometimes” to “often.” In item 6, only b_4_ is positive. This suggests that lower latent trait or theta should endorse Likert-type response options—from “never” to “sometimes.” Scale information curve (Supplementary Figure 1) provides an understanding of the information provided by the SAVE-6. From this curve, this scale provides more information about people between −1.5 and 0.75 θ level. There are double peaks in the curve, which might be due to the polytomous nature of the data.

### Reliability of the SAVE-6 Scale and Evidence Based on Relations to Other Variables

The SAVE-6 scale showed good internal consistency (Cronbach's alpha = 0.827, McDonald's omega = 0.834, split-half reliability [odd-even] = 0.895). A Cronbach's alpha if item dropped were 0.777 ~ 0.848. Mean inter-item correlation of this scale (0.444) was in recommended rage (between 0.15 and 0.50). This scale also had good discrimination power (Ferguson delta = 0.99). This scale also good IRT reliability (0.880) and rho coefficient (0.836). The SAVE-6 score was significantly correlated with Goldberg anxiety score (*r* = 0.434, *p* < 0.001), depression score (*r* = 0.193, *p* = 0.043), and PSQI score (*r* = 0.262, *p* = 0.002). However, the correlation between the SAVE-6 and Goldberg depression scale disappear, if we define the significance level as two-tailed *p* < 0.0167 (as 0.05/3) due to multiple comparison. The SAVE-6 score was significantly higher among participants who were assessed as having anxiety symptoms [Goldberg anxiety score ≥ 4, *t*_(133)_ = 3.368, *p* < 0.001] and depression [Goldberg depression score ≥ 2, *t*_(133)_ = 2.647, *p* < 0.001]. Furthermore, the SAVE-6 scale score was higher among younger individuals (20–39 years old) vs. older individuals (over 40 years old) [*t*_(133)_ = 3.131, *p* = 0.002], nurses vs. physicians [*t*_(133)_ = 2.296, *p* = 0.023], and those who had symptoms of COVID-19 infection [*t*_(133)_ = 2.621, *p* = 0.01]. However, the SAVE-6 score was not significantly higher across gender [*t*_(133)_ = 0.851, *p* = 0.396], living with partners [*t*_(133)_ = 0.605, *p* = 0.546], or having family members who were infected with COVID-19 [*t*_(133)_ = 0.569, *p* = 0.570)].

## Discussion

In this study, we found that the SAVE-6 scale, derived from the SAVE-9 scale for the general population, can be a reliable and valid rating scale for measuring the viral anxiety of healthcare workers, specifically in response to the viral epidemic. In this sample of Spanish healthcare workers, we observed that the SAVE-6 scale, a rating scale used to assess the anxiety response of the general population (17–19), can also be applied to measure the anxiety response of healthcare workers, specifically to the viral epidemic.

The original SAVE-9 scale was developed to assess work-related stress and anxiety response to the viral epidemic during this COVID-19 pandemic (11). It was clustered into two factors: factor I (item 1, 2, 3, 4, 5, and 8) and factor II (item 6, 7, and 9), and this clustering was parallel to the other validation studies of Italian, Japanese, and Turkish versions of the SAVE-9 scale. However, that clustering was not observed in other languages such as Russian (12) and German (16); factor I (item 2, 3, 4, and 8) and factor II (item 1, 5, 6, 7, and 9). The SAVE-6 was originally derived from the SAVE-9 scale; all items of SAVE-6 were selected to measure the anxiety response of healthcare workers. Previously it was applied to medical students (20) and public workers (21) whose social role is close to healthcare workers by serving patients or clients at higher risk of viral infection. Applicability of the SAVE-9 Scale failed for public workers (22). Rather, Chung et al. (21) observed that the SAVE-6 could be applied to measure the anxiety response of public workers.

The construct validity of the SAVE-6 scale is better in the Spanish healthcare workers sample compared to those from our previous studies on the general population (17, 18), public workers (21), or medical students (20). The reliability of the SAVE-6 scale, based on Cronbach's alpha and McDonald's omega, was comparable to that of previous studies. Unfortunately, we could not compare the cut-off point of the SAVE-6 scale among healthcare workers in Spain with those of previous studies since we did not apply the Generalized Anxiety Disorder-7 items (GAD-7) scale in this study. Previously, 12 ~ 16 points were defined as a cut-off score of SAVE-6 in accordance with 5 points on the GAD-7 scale (mild degree of general anxiety) from the Receiver-Operating Curve analysis results. It is important to maintain the compatibility of rating scale across cultures, groups, or countries. Since we developed the SAVE-9 or SAVE-6 scale as a measuring tool in accordance with mild degree of generalized anxiety (GAD-7 ≥ 5), we decided to define the cut-off score in accordance with GAD-7 scale in the future study.

During the COVID-19 pandemic, healthcare workers may suffer from related anxiety symptoms. However, such symptoms are non-pathological unlike the pathological symptoms of Generalized Anxiety Disorder (36). Nevertheless, it is important to monitor and manage the psychological stress of healthcare workers not only for their own safety but also for that of their patients (37, 38). Therefore, the SAVE-6 scale will be beneficial in measuring and screening healthcare workers' viral anxiety, which is often neglected. We can (1) measure anxiety responses using a brief and short rating scale, and (2) compare the anxiety response of healthcare workers to the viral epidemic with various population groups such as the general population (17, 18), public workers (21), medical students (20), or cancer patients (22). It will be helpful to develop a psychological support system for specific groups after comparing the level of anxiety during a pandemic. However, applying the SAVE-6, rather than the SAVE-9, scale to healthcare workers, might lead to a decrease in the value of “specificity to the HCW.” The items of factor II of the original SAVE-9 scale (Item 6—Do you feel skeptical about your job after going through this experience? Item 7—After this experience, do you think you will avoid treating patients with viral illness? Item 9—Do you think your colleagues would have more work to do due to your absence from a possible quarantine and might blame you?) were highly specific to work-related stress. We now try to apply factor II as a brief measure for work-related stress. We can guess the value of “specificity to healthcare workers” of the SAVE-9 come from the SAVE-3 rather than SAVE-6. The items of the SAVE-6 seem non-specific to healthcare workers but specific to the viral epidemic. Further study will explore the factorial validity of the SAVE-3 scale as a tool for measuring work-related stress of healthcare workers related to the viral epidemic (39). Therefore, if we aimed to measure the anxiety response of healthcare workers specifically to the viral epidemic, applying the SAVE-6 might be enough.

This study had several limitations. First, one of the limitations could be that it was an online survey. Due to the pandemic, we conducted an online survey rather than face-to-face interviews. It can lead to biased data. Second, a higher proportion of depression (82.2%) may influence the results. We should consider the possibility that the depression subcategory of the Goldberg scale can be highly sensitive and less specific for assessing depression. We can expect 50% of possible depression in the original literature using the cut-off point (25). Third, sampling bias should be considered. As previously addressed, the skewness and kurtosis of item 7 are relatively high. Further studies need to be conducted to explore whether the applicability of the Spanish version of the SAVE-9 scale to healthcare workers can be supported by the CFA in other Spanish healthcare workers' samples. Last, we could not assess past history of psychiatric disorder or current psychiatric problems of participants. It might influence the understanding of the results of this study.

In conclusion, the Spanish version of SAVE-6 is a reliable and valid rating scale to assess the anxiety response of healthcare workers specifically to the viral epidemic as a brief measure during this COVID-19 pandemic. It showed that we can apply the brief version of SAVE-9 to HCW with good validity and reliability. Applying the SAVE-6 to HCW may lose the value of “specificity to the HCW,” but we can keep the value of “specificity to the viral epidemic” of the scale. However, we believe that these results might not decrease the value of the SAVE-9 scale for the HCW, since the score might have been influenced by the prolonged COVID-19 pandemic and the healthcare workers' call of duty for the patients.

## Data Availability Statement

The raw data supporting the conclusions of this article will be made available by the authors, without undue reservation.

## Ethics Statement

The study protocol was approved by the Institutional Review Board of Grupo Hospitalario QuirónSalud-Cataluña (2020/52-MSU-TEK). The patients/participants provided their written informed consent to participate in this study.

## Author Contributions

MM-C and SC conceived the study. MM-C and JA-T obtained ethics approval, recruited participants and obtained data. MM-C, SC, and OA organized the database and performed statistical analyses. All authors contributed to writing a draft, revision, and final approval of the submitted version to be published.

## Funding

MM-C was supported by a research grant of the Spanish Sleep Society (SES).

## Conflict of Interest

The authors declare that the research was conducted in the absence of any commercial or financial relationships that could be construed as a potential conflict of interest.

## Publisher's Note

All claims expressed in this article are solely those of the authors and do not necessarily represent those of their affiliated organizations, or those of the publisher, the editors and the reviewers. Any product that may be evaluated in this article, or claim that may be made by its manufacturer, is not guaranteed or endorsed by the publisher.
