# Supplementary figures and images for: Validity and Reliability of the Stress and Anxiety to Viral Epidemics-6 (SAVE-6) Scale to Measure Viral Anxiety of Healthcare Workers in Spain During the COVID-19 Pandemic
Source: Front Psychiatry. 2022 Feb 1;12:796225. doi: 10.3389/fpsyt.2021.796225 (PMC8846288; doi:10.3389/fpsyt.2021.796225)

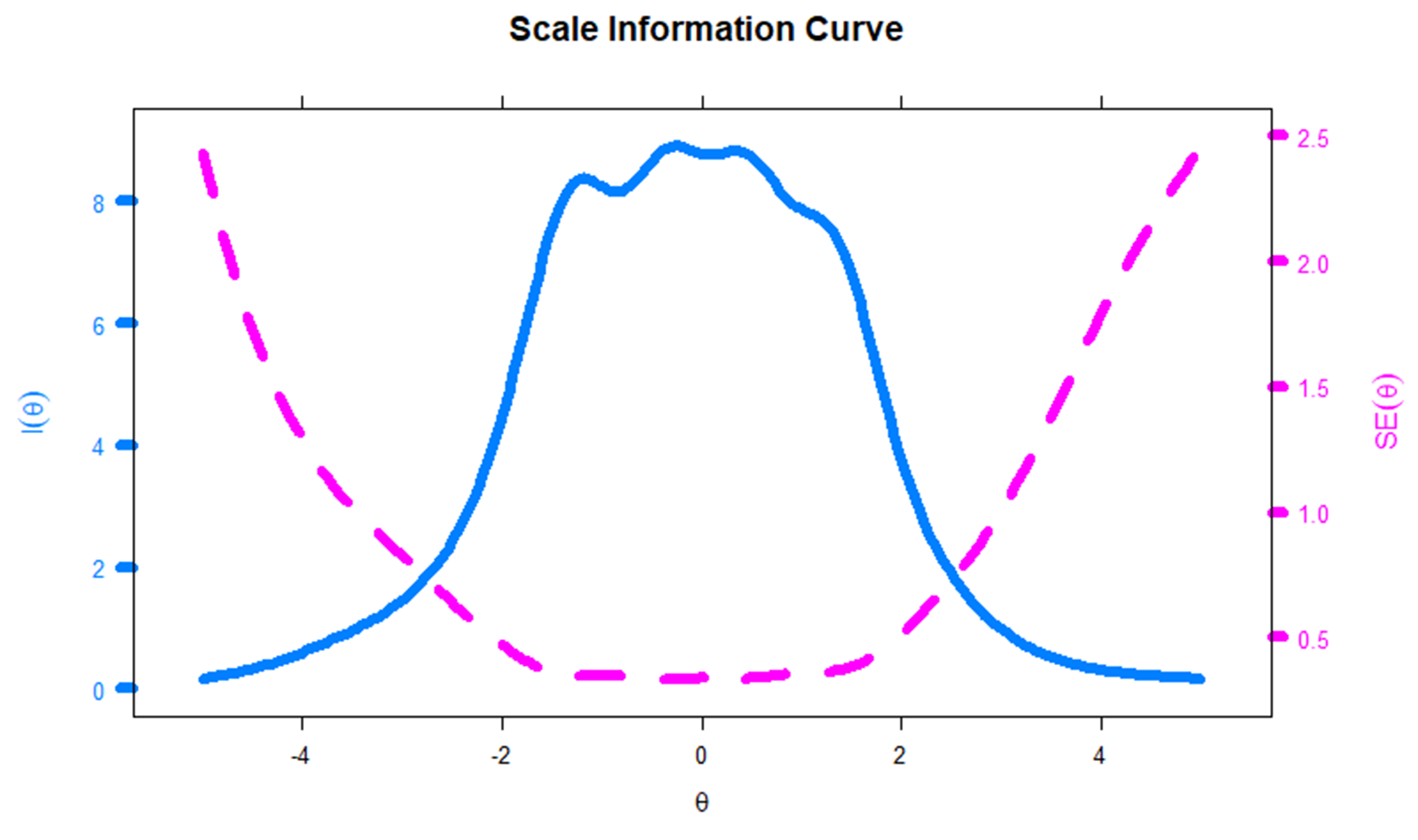

Supplement: Supplementary file 1 [file Image_1.PNG]
