# Supplementary material for: Validity and Reliability of the Stress and Anxiety to Viral Epidemics-6 (SAVE-6) Scale to Measure Viral Anxiety of Healthcare Workers in Spain During the COVID-19 Pandemic
Source: Front Psychiatry. 2022 Feb 1;12:796225. doi: 10.3389/fpsyt.2021.796225 (PMC8846288; doi:10.3389/fpsyt.2021.796225)
Supplement: Supplementary file 2 [file Data_Sheet_1.docx]

Supplementary Material

# Supplementary Table 1. H-coefficients of items, local dependance G2 p-values, and monotonicity statistics of the SAVE-6

|  | ***H-*coefficients** | **Monotonicity** | | | | **Local dependance G^2^ p-values** | | | | |
| --- | --- | --- | --- | --- | --- | --- | --- | --- | --- | --- |
|  |  | **#ac** | **#vi** | **#zsig** | ***Crit*** | **Item1** | **Item2** | **Item3** | **Item4** | **Item5** |
| **Item1** | .58 | 3 | 0 | 0 | 0 |  |  |  |  |  |
| **Item2** | .76 | 3 | 0 | 0 | 0 | .270 |  |  |  |  |
| **Item3** | .76 | 3 | 0 | 0 | 0 | .270 | .274 |  |  |  |
| **Item4** | .61 | 3 | 0 | 0 | 0 | .270 | .270 | .270 |  |  |
| **Item5** | .50 | 3 | 0 | 0 | 0 | .270 | .270 | .270 | .270 |  |
| **Item6** | .64 | 2 | 0 | 0 | 0 | .270 | .270 | .270 | .270 | .270 |
| ac = active comparison, vi = violation, zsig = significant violation  Notes: p-values adjusted for false discovery rate (FDR) | | | | | | | | | | |

# Supplementary Table 2. Item misfit information, slope, and threshold parameters of the SAVE-6

| Items | Item fits | | | Slope parameter (α) | Threshold parameter (b) | | | |
| --- | --- | --- | --- | --- | --- | --- | --- | --- |
|  | S-χ^2^ | df | p-value |  | b_1_ | b_2_ | b_3_ | b_4_ |
| Item 1 | 17.504 | 18 | .560 | 1.626 | -3.269 | -1.529 | .001 | 1.497 |
| Item 2 | 26.244 | 16 | .306 | 3.254 | -1.173 | -.300 | .540 | 1.415 |
| Item 3 | 15.490 | 17 | .560 | 3.103 | -1.421 | -.403 | .359 | 1.211 |
| Item 4 | 19.312 | 18 | .560 | 1.944 | -1.327 | .076 | .561 | 2.129 |
| Item 5 | 34.373 | 28 | .560 | .752 | -.274 | 1.353 | 2.921 | 4.358 |
| Item 6 | 15.604 | 17 | .560 | 2.085 | -2.051 | -1.465 | -.474 | .370 |
| Notes: p-values adjusted for false discovery rate (FDR) | | | | | | | | |
